# Effects of progestin-only contraceptives on the phenotype and function of female reproductive tract CD4+ and CD8+ T cells

**DOI:** 10.1186/1471-2334-14-S3-O21

**Published:** 2014-05-27

**Authors:** Uma Shanmugasundaram, JW Critchfield, J Pannell, J Perry, WC Greene, L Giudice, K Smith-McCune, RM Greenblatt, BL Shacklett

**Affiliations:** 1 University of California, Davis, USA; 2 University of California, San Francisco, USA; 3 Gladstone Institute of Virology and Immunology, San Francisco, CA, USA

## Background

Use of progestin-only contraceptives may enhance the susceptibility of the female reproductive tract (FRT) to HIV infection. We assessed the effects of progestin-only contraceptives, including Depo-Provera (DMPA), and the levonorgestrel intra-uterine system (LNG iUS) on endometrial and endocervical mucosa of premenopausal women.

## Methods

Participants using either DMPA (n=15) or the LNG iUS (n=19) for at least 3 months and women using no hormonal contraceptives (n=24) were recruited. Endocervical curettage, endocervical cytobrush, and endometrial biopsy were obtained from all women. Expression of T cell activation markers, memory/effector differentiation markers and HIV co receptors were assessed by multiparameter flow cytometry. CD4+ and CD8+ T cells producing CD107a, IL-10, IL-2, IFNγ, MIP1β and IL-17 were measured after stimulation with PMA/ionomycin or staphylococcal enterotoxin B (SEB).

## Results

Endometrial CD4+ and CD8+ T cells and endocervical CD8+ T cells were highly activated in women using LNG iUS as compared to controls. Compared to DMPA, LNG iUS resulted in increased activation of endocervical curettage CD4+ T cells. CXCR4+CCR5+CD4+ T cells in the endometrium and curettage were increased in women using LNG iUS as compared to controls. The percentage of endocervical central memory CD4+ and CD8+ T cells were decreased in LNG iUS recipients. Following SEB stimulation, endometrial CD4+ T cells responded with production of IL-10, IL-2 and IL-17. CD8+ T cells produced elevated percentages of IL-10 in women using LNG iUS compared to control women.

## Conclusion

LNG iUS and DMPA affected endocervical and endometrial T cell phenotype and responsiveness to polyclonal stimulation. Further studies are warranted to clarify the effects of contraceptive products on upper FRT immune cells and HIV susceptibility.

